# Cause-specific heat-related mortality in Rio de Janeiro city: Comparing exposure metrics and the role of exposure duration

**DOI:** 10.1097/EE9.0000000000000473

**Published:** 2026-05-28

**Authors:** João Henrique de Araujo Morais, Débora Medeiros de Oliveira e Cruz, Valeria Saraceni, Caroline Dias Ferreira, Gislani Mateus Oliveira Aguilar, Oswaldo Gonçalves Cruz

**Affiliations:** a Sergio Arouca National School of Public Health, Oswaldo Cruz Foundation (Fiocruz), Rio de Janeiro, Brazil; b Rio de Janeiro Municipal Health Secretariat (SMS), Rio de Janeiro, Brazil; c Medical Education Institute (IDOMED), Rio de Janeiro, Brazil; d Program of Scientific Computing (PROCC), Oswaldo Cruz Foundation (Fiocruz), Rio de Janeiro, Brazil

**Keywords:** extreme heat, mortality, epidemiology, elderly, Brazil

## Abstract

Under a climate change scenario, extreme heat episodes show an increase in frequency and intensity, with a scaling impact in Latin American cities. Recently, Rio de Janeiro City developed its heat protocol, using the amount of hours spent over specific heat thresholds as its trigger metric. This study gathers mortality data by 17 causes of death in Rio, over a 12.5-year period (2012–2024). We use Distributed Lag Nonlinear Models to assess the relationship between heat exposure and mortality among younger (0–64 years) and elderly (65+ years) age groups, comparing temperature and heat index as exposure metrics. Additionally, we evaluate whether including measures of exposure duration contributes to the explanation of heat-related mortality. In the study period, there were 466,121 deaths in the city from natural causes. Of the 17 causes of death, six showed a significant association with heat among the younger age group (0–64), while 13 were significant among the elderly. Particularly elevated relative risks were observed for deaths due to diabetes, Alzheimer’s and dementia, hypertensive diseases, urinary tract infections, and deaths by undetermined causes. Even though no clear conclusion was reached on the temperature and heat index comparison, the inclusion of exposure duration showed a relevant additional effect for explaining mortality across broader cause groups. The results characterize cause-specific heat-related mortality in Rio and show potential on the use of exposure period metrics to better understand heat-mortality relationships and guide definitions for heat warning systems.

**What this study adds**

This study contributes to the characterization of cause-specific heat-related mortality in a Latin American city, understanding the most affected causes across lag structures and age groups. Besides adding to the literature of exposure metric comparison in such studies, particularly innovative evidence is provided from the inclusion of exposure duration variables. The findings suggest that, beyond traditionally used metrics like daily averages, accounting for the duration of exposure to extreme heat can improve the understanding of heat-mortality associations and guide definitions for heat protocols such as Rio’s recent one.

## Introduction

As Earth reaches its warmest years between 2023 and 2025,^1–3^ heat wave episodes are becoming more frequent and intense across the globe.^4–7^ This temperature increase and a considerable portion of its related mortality have been shown to be due to human action^8,9^ and projections show that extreme events such as heat waves will get longer and even more frequent in the future.^9,10^ The health burden caused by heat is well documented in the literature. Exposure to extreme temperatures can affect the pathophysiological mechanisms of thermoregulation, distribution of body flow, and sweating mechanisms.^11,12^ Studies around the world have extensively shown associations of heat waves with cardiovascular and respiratory diseases,^13–16^ renal diseases,^17–19^ mental health^5,20^ and pregnancy outcomes.^21,22^ Certain population groups are known to be at increased risk, such as older populations and those with coexisting health conditions.^12,23–26^ Disparities in risk are also shown to exist across gender and race.^5,27^

There has been an increasing attention to heat effects in health in Latin American countries,^16,28^ as the Latin population is aging^29^ and the number of heat-related extreme episodes in the region is scaling.^9,30^ In Brazil, most single-city studies concentrate in the cities of São Paulo^24,31,32^ and Rio de Janeiro.^25,33^ Recently, Moraes et al^24^ showed a higher risk for cardiovascular and respiratory mortality under different definitions of heat waves among the elderly population in São Paulo. Silveira and collaborators showed similar results for the city of Rio de Janeiro^25^ and also for 32 municipalities in the Brazilian Amazon.^34^ Santos et al^5^ showed an increase in the number, intensity and duration of heatwaves in the 14 most populous metropolitan regions of Brazil, and estimated 48,075 heat-related excess deaths in the period of 2000–2018.

As cities have dealt with heat waves for some time, heat warning systems (HWS) have been implemented around the world over the last decades,^35–38^ with the goal of warning, communication, and protection of the population. Brazil faced one of its worst heat waves in history in November 2023,^39^ which resulted in a remarkable fan’s death due to heat exhaustion during a concert in Rio de Janeiro.^40^ In June 2024, Rio launched a protocol for dealing with extreme heat in the city.^41^ Differently from existing HWS, its triggers are based upon the event duration and the amount of time the population is exposed to high heat indices within a day. However, epidemiological literature still lacks studies that incorporate exposure duration when evaluating heat-health associations.

The present study aims to quantify associations between heat exposure and cause-specific mortality across 17 different causes of death in Rio de Janeiro city (RJC), among younger and older age groups. It also intends to assess whether the heat index (HI), frequently used as a composite index for thermal comfort, offers any improved performance compared to temperature in explaining heat-related mortality among the included causes. Finally, measures of exposure duration are also incorporated in order to evaluate whether prolonged periods of heat exposure imply additional risk when dealing with heat-mortality associations.

## Methods

### Data and variables

#### Mortality data

Mortality data were retrieved from the Brazilian Mortality Information System (Sistema de Informações de Mortalidade), referring to deaths that occurred from 2012 to the first 6 months of 2024. We filtered deaths of residents of RJC (CO_MUN_RES = 330455). The ICD-10 field of underlying cause of death was used to classify deaths into 17 groups of causes possibly related to heat exposure. These groups were defined based on a CDC’s Excess Mortality Study.^42^ Additional groups for urinary tract deaths and unspecified deaths were included. Finally, analyses were also run for two aggregated groups: selected deaths (all causes previously included) and natural causes of death (all deaths except external causes). The groups and their corresponding ICD-10 codes are summarized in Table 1. Daily death counts by cause group were aggregated considering two age groups: 0–64 years old and 65+.

**Table 1.** Causes of death groups included in the study, and respective ICD-10 codes

Cause of death group	ICD-10 codes
Influenza and Pneumonia	J09–J18
Chronic lower respiratory diseases	J40–J47
Other diseases of the respiratory system	J00–J06, J20–J39, J60–J70, J80–J86, J90–J96, J97–J99, R09.2, U04
Hypertensive diseases	I10–I15
Ischemic heart disease	I20–I25
Heart failure	I50
Cerebrovascular diseases	I60–I69
Other diseases of the circulatory system	I00–I09, I26–I49, I51, I52, I70–I99
Malignant neoplasms	C00–C97
Alzheimer disease and dementia	G30, G31, F01, F03
Diabetes	E10–E14
Renal failure	N17–N19
Sepsis	A40–A41
Urinary tract disorder	N39
Unspecified causes	R00–R99
Selected causes	All of the above
Natural causes	All except external causes (T00–Y99)

Adapted from Centers for Disease Control and Prevention.^42^

#### Climate data

Hourly temperature (T) and relative humidity (H) data were retrieved for 16 weather stations, from three different sources: Alerta Rio System (https://www.sistema-alerta-rio.com.br/, seven stations), Brazilian National Meteorological Institute (Instituto Nacional de Meteorologia, four stations), and Aeronautics Command Meteorology Network (Rede de Meteorologia do Comando da Aeronáutica, five stations). Stations’ locations are represented in Supplementary Figure 1; https://links.lww.com/EE/A429. HI following Steadman^43^ and the operational formulation from the U.S. National Oceanic and Atmospheric Administration(NOAA, https://www.wpc.ncep.noaa.gov/html/heatindex_equation.shtml) was calculated, and the hourly median HI among all available stations was considered for the analysis. This is the procedure already done in Rio’s heat monitoring, in an attempt to obtain a summary metric for the municipality, disregarding extreme values from specific stations.^41^

Therefore, for each day in the study period (Jan/2012–Jun/2024), daily average temperature (T_med_) and heat index (HI_med_) were obtained, calculated as the mean of the median hourly T and HI among the available stations.

#### Exposure period

Other than summary metrics such as T_med_ or HI_med_, RJC’s heat protocol bases its triggers on the amount of hours in a given day spent with HI values above a certain threshold.^41^ Therefore, this study intended to analyze whether there is an additional effect of days with prolonged periods of exposure to extreme heat that are not fully captured by daily averages. Therefore, for each day, the amount of hours spent (0–24) with an HI above the hourly HI 90th, 95th, and 97.5th quantiles were calculated. Finally, the hour counts were summarized as indicator variables (0/1), representing normal (below or equal 90th quantile of the hour counts distribution) or high (above 90th quantile) period of exposure. In the rest of this paper these variables are called “Hours above Q90,” “Hours above Q95” and “Hours above Q975” for simplification.

### Statistical analyses

Daily mortality outcomes were modeled using Generalized Additive Models, with cross-basis functions from Distributed Lag Nonlinear Models (DLNM).^44^ To determine a death count baseline for each cause, smooth terms for long-term trend and seasonality were included and are detailed subsequently. Day of week (dow) was included as a covariate as it can be a source of confounding (holidays were considered as “Sunday”).^14,45^ Even though air pollution is frequently considered a relevant variable in the heat-mortality relationship as well^46–48^ it could not be included in the analyses due to low data availability on PM 2.5 and PM 10 emissions for the study period in the city of Rio.

#### Model structure

Summary statistics (mean, median, variance, variance/mean ratio, and percentage of null counts) were calculated for each cause of death and age group series. Series with a daily median count equal to or lower than 1 or with a null percentage greater than 20% were not considered for modeling due to low death counts. The series with a variance/mean ratio higher than 1.6 were modeled with a quasipoisson likelihood, and a Poisson likelihood was used otherwise.

To capture long-term trends, a penalized cubic regression spline with k=6 knots was used. This provides sufficient flexibility to capture trends in mortality over the study years, and also account for a potential change in the mortality pattern during the COVID-19 pandemic period. For intrayear seasonality, a cyclic cubic regression spline was fitted over the day-of-year (doy) variable, that ranges from 1 to 366. A k = 6 number of knots was used to allow for two yearly mortality peaks that usually occur.

#### Distributed lag nonlinear models

The base model defined above was extended to include the exposure variables of interest, using the crossbasis() function from R package {dlnm}^49^ to account for nonlinearity and lagged effects.^50^ Lag periods up to 10 days were considered, motivated by previous literature.^14^ For T_med_ and HI_med_ a natural spline with df = 4 was used, given that a response curve in the “U” shape is expected (higher mortality risk in cold and warm extremes). Since the study’s interest lies on the understanding of the heat-related mortality thresholds, knots were placed at the 10th, 75th, and 90th quantiles as done in similar studies.^16,51^ For the lag term, a natural spline was also used with df = 3. The knots were not spaced in the log scale as it is usually done^52^; instead, they were placed at lags 2, 5, and 8 with the justification that for some causes, a more acute effect is expected for later lags.^25,53^ Finally, to test whether the exposure period to high HI would bring any additional benefit to the explanation of the outcomes, the hours above Q90, Q95, and Q975 variables were added to the previously fitted models with T_med_ and HI_med_. Since they are Boolean variables (0/1), a linear function was used.

To collect the results, predictions for the DLNMs were extracted using the mean as the centering value for T_med_ and HI_med_ models, and 0 for the hours indicator variables. Models for the same cause and age group were compared in terms of the Akaike Information Criterion (AIC) (AIC, in case of Poisson), or "quasi-AIC" (qAIC, in case of quasipoisson). The values for T_med_ and HI_med_ that represented the first point that a significant increase in mortality risk is seen (when the lower bound of the confidence interval [CI] is greater than 1) were calculated and shown as quantiles. Estimated relative risks (RR’s) for the 75th and 90th quantile of the exposure distributions (where knots were placed) were also calculated. Finally, exposure-response curves were obtained for all lags and individually for each lag to determine which causes are most affected by age group, and the lag interval (0–10) over which the RRs are highest.

### Software

All analyses were run under the “R” environment, in 4.4.1 version.^54^ Packages “mgcv” (1.9.1) and “dlnm” (v 2.4.7) were used. All the developed code is available on GitHub: https://github.com/joaohmorais/EE_submission_heat_mortality_RJC/.

## Results

During the study period, 390,244 deaths from selected causes and 466,121 deaths from natural causes were registered in RJC. Table 2 describes summary metrics for death counts due to the 17 cause of death groups included in the study in each age group considered. Malignant neoplasms and ischemic heart disease (IHD) ranked as causes with the highest counts for both age groups (not considering the aggregated selected and natural deaths groups), with Influenza and Pneumonia also ranking among the elderly. Renal failure deaths could not be modeled in either age group due to low daily counts. For the younger group (0–64), only nine of the 17 causes had sufficient values for modeling. Summary metrics for the exposure variables are exhibited in Table 3. The mean for the city during the study period was 24.19 °C and 25.14 °C for T_med_ and HI_med_, respectively, with the latter showing higher variance and concentration of higher values, as expected. A histogram that shows a longer tail for the HI_med_ distribution can be seen in Supplementary Figure S3; https://links.lww.com/EE/A429. The hourly variables (number of hours in a day where an HI greater than its hourly 90th, 95th, and 97.5th quantile is registered) show that counts are mostly zero, but can be as high as 23, 17, and 15 hours in a day for hours above quantile 90, 95, and 97.5, respectively. Supplementary Figure 4; https://links.lww.com/EE/A429, shows that days with similar T_med_ can have different periods of exposure to high HI quantiles. An example is December 20th, 2017, and March 5th, 2024: while both days had similar T_med_ (≈27.55 °C), the first had only 2 hours above the 90th quantile of HI, while the latter had 12.

**Table 2.** Summary metrics (total in the study period, mean, median, variance, variance over mean ratio, and percent of days with zero counts) for daily death counts, by cause of death and age group

Cause of death	Total deaths	Mean	Median	Variance (Var.)	Var/Mean.	Percent zero
Age 0–64						
Alzheimer’s and dementia	276	0.06	0	0.06	1	94.15
Cerebrovascular diseases	12,593	2.76	3	2.87	1.04	6.81
Chronic lower respiratory diseases	3,293	0.72	1	0.76	1.05	49.13
Diabetes	8,710	1.91	2	1.99	1.04	15.27
Heart failure	3,098	0.68	0	0.69	1.02	51.06
Hypertensive diseases	7,105	1.56	1	1.64	1.06	22.52
Influenza and pneumonia	10,046	2.2	2	2.42	1.1	12.18
Ischemic heart disease	18,896	4.14	4	4.65	1.12	1.66
Malignant neoplasms	43,821	9.6	9	9.86	1.03	0.02
Other diseases of the circulatory system	13,034	2.86	3	3.33	1.17	7.38
Other diseases of the respiratory system	4,714	1.03	1	1.17	1.13	37.42
Renal failure	2,541	0.56	0	0.57	1.02	57.26
Sepsis	4,927	1.08	1	1.16	1.07	34.94
Undetermined deaths	13,617	2.98	3	3.88	1.3	6.59
Urinary tract infections	2,118	0.46	0	0.47	1.02	63.11
Selected causes	148,342	32.5	32	38.05	1.17	0.00
Natural causes	208,949	45.77	44	121.95	2.66	0.00
Age 65+						
Alzheimer’s and dementia	13,154	2.88	3	3.08	1.07	6.31
Cerebrovascular diseases	34,799	7.62	7	8.22	1.08	0.07
Chronic lower respiratory diseases	14,465	3.17	3	3.62	1.14	5.26
Diabetes	22,408	4.91	5	5.31	1.08	0.99
Heart failure	10,152	2.22	2	2.4	1.08	11.72
Hypertensive diseases	24,484	5.36	5	7.3	1.36	0.90
Influenza and pneumonia	46,555	10.2	10	14.34	1.41	0.02
Ischemic heart disease	43,817	9.6	9	11.77	1.23	0.00
Malignant neoplasms	74,789	16.38	16	17.49	1.07	0.00
Other disease of the circulatory system	28,678	6.28	6	7.85	1.25	0.35
Other diseases of the respiratory system	12,917	2.83	3	3.31	1.17	6.29
Renal failure	6,175	1.35	1	1.4	1.04	26.29
Sepsis	19,433	4.26	4	4.96	1.17	2.12
Undetermined deaths	22,568	4.94	4	11.03	2.23	2.19
Urinary tract infections	17,357	3.8	4	4.53	1.19	3.42
Selected causes	390,147	85.46	85	172.28	2.02	0.00
Natural causes	466,007	102.08	99	474.14	4.64	0.00

**Table 3.** Summary metrics for temperature variables

Variable	n (%)	Min.	Q10	Mean	Median	Q90	Q95	Q975	Q99	Max.	Var.
Daily metrics											
Tmed (°C)	-	15.18	20.41	24.19	24.1	28.17	29.09	29.7	30.4	32.1	8.59
HI_med_ (°C)	-	14.97	20.53	25.14	24.69	30.38	31.71	32.67	33.82	39.69	14.05
Hourly variables											
Hourly T (°C)	-	11	19.32	24.19	24	29.7	31.46	33	34.5	39.85	15.7
Hourly HI (°C)	-	11.01	19.63	25.14	24.39	32.14	34.55	36.39	38.15	49.08	23.36
Amount of hours											
Above hourly Q90	-	0	0	2.4	0	9	11	12	15	23	16.03
Above hourly Q95	-	0	0	1.2	0	6	8	9	11	17	7.32
Above hourly Q975	-	0	0	0.6	0	2	5	7	9	15	3.36
Hours above Q90 (HI = 32.14 °C)											
High (above 9 hours–Q90)	401 (8.78)	-	-	-	-	-	-	-	-	-	-
Low	4,164 (91.21)	-	-	-	-	-	-	-	-	-	-
Hours above Q95 (HI = 34.55 °C)											
High (above 6 hours–Q90)	381 (8.34)	-	-	-	-	-	-	-	-	-	-
Low	4,184 (91.65)	-	-	-	-	-	-	-	-	-	-
Hours above Q975 (HI = 36.39 °C)											
High (above 2 hours–Q90)	442 (9.68)	-	-	-	-	-	-	-	-	-	-
Low	4,123 (90.32)	-	-	-	-	-	-	-	-	-	-

For continuous variables, minimum and maximum values, mean, and quantiles are presented; for categorical variables, absolute (n) and relative (%) frequencies are shown.

**Figure 1.**
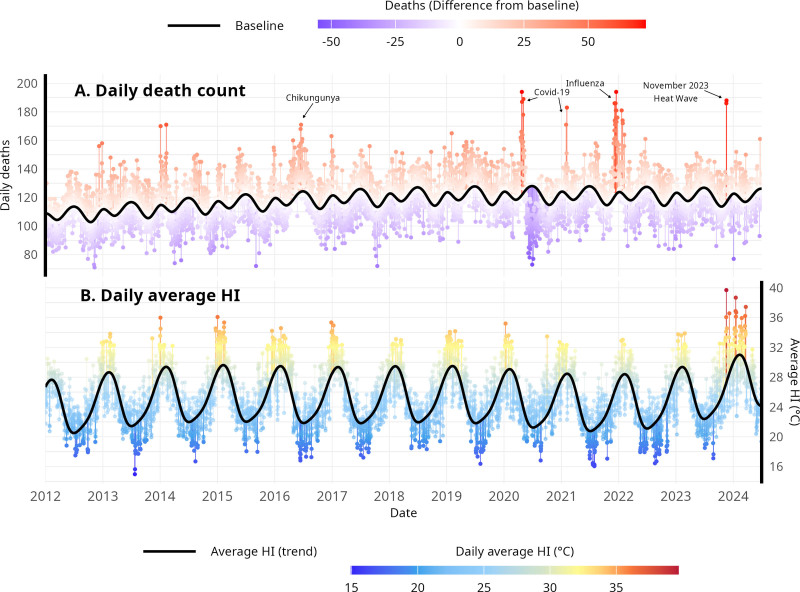
Baseline for daily deaths (solid line) for all ages in selected causes, and difference between observed and baseline (A), and daily average heat index trend (solid line) and observed values (B), for RJC, Jan/2012–Jun/2024.

Observed death counts from selected causes and its model-estimated baseline are shown in Figure 1A, along with HI_med_ trends (Fig. 1B). We can describe the seasonality of deaths with two yearly peaks: a higher one occurring in the fall/winter period (April–June) and the other during the summer period (December, January). This seasonality effect by cause of death can be seen in Supplementary Figure S6; https://links.lww.com/EE/A429, where the estimated effects for the seasonality term are plotted. As deaths due to coronavirus infection (ICD-10 codes B34.2 and U07.1) were not included among selected causes, the death trend during the COVID-19 period showed a slight decrease in the death counts. Some notorious excess death periods can be named: in the fall/mid-year of 2016, a mortality burden due to the Chikungunya epidemic in RJC that year was observed.^55^ In 2020 and 2021, three mortality peaks can be pointed: the first in early 2020, after COVID-19 arrival in the country; the second in the period of Gamma (P.1) variant predominance, in early 2021; and the latter during the Influenza A H3N2 outbreak in late 2021.^56^ Finally, a considerable peak is then observed in November 2023, in two following days: November 18th and 19th–the last 2 days of November 2023 were a remarkable heat wave.

### Most affected causes, by age group

Table 4 summarizes results from the DLNM models for T_med_ and HI_med_ exposures. “T_Risk” and “HI_Risk” represented in the table correspond to the T_med_ and HI_med_ values for which the first significant increase in mortality risk (lower bound of the 95% CI greater than 1) was seen. RR’s at Q75 and Q90, where the knots of the spline were positioned, are also shown. Significant mortality increases for the 0–64 age group were observed for all modeled causes except cerebrovascular diseases, influenza and pneumonia and malignant neoplasms. Diabetes and undetermined deaths had the highest significant RR at Q90: 1.21 and 1.15 for T_med_, respectively. Among the elderly group (65+), only chronic lower respiratory diseases, heart failure, and malignant neoplasms showed no association; the other 13 death causes had a significant mortality risk increase at some point. The highest RR’s at Q90 were observed for undetermined deaths (1.42 for HI_med_), urinary tract infections (1.33 for HI_med_) and Alzheimer’s and dementia (1.31 for HI_med_).

**Table 4.** Model-estimated relative risks (RRs) for T_med_ and HI_med_: Temperature/HI when a significant RR above 1 is reached (T_Risk/HI_Risk); RRs at quantiles 75 (Q75) and 90 (Q90) of the exposure, by death cause and age group

Cause of death	Tmed			HImed		
	T_Risk	RR at Q75	RR at Q90	HI_Risk	RR at Q75	RR at Q90
0–64						
Cerebrovascular diseases	-	0.98 (0.94–1.02)	1.01 (0.90–1.13)	-	0.98 (0.95–1.02)	1.02 (0.92–1.12)
Diabetes	26.8 °C (Q79)	1.05 (0.99–1.11)	**1.21 (1.05–1.40**)	29.5 °C (Q85)	1.03 (0.98–1.09)	**1.16 (1.03–1.32**)
Influenza and pneumonia	-	1.01 (0.95–1.08)	0.95 (0.83–1.10)	-	1.01 (0.95–1.07)	0.99 (0.87–1.12)
Ischemic heart disease	28.5 °C (Q92)	1.02 (0.99–1.06)	1.09 (0.99–1.19)	28.5 °C (Q79)	1.02 (0.99–1.06)	**1.10 (1.02–1.19**)
Malignant neoplasms	-	0.98 (0.96–1.01)	0.95 (0.90–1.01)	-	0.98 (0.96–1.01)	0.97 (0.92–1.03)
Other disease of the circulatory system	29.0 °C (Q95)	1.02 (0.97–1.06)	1.04 (0.93–1.16)	31.7 °C (Q95)	1.04 (1.00–1.07)	1.07 (0.97–1.17)
Undetermined deaths	28.0 °C (Q89)	1.02 (0.97–1.08)	**1.15 (1.02–1.30**)	29.7 °C (Q87)	1.03 (0.98–1.08)	**1.15 (1.03–1.28**)
Selected causes	29.0 °C (Q95)	1.00 (0.99–1.02)	1.02 (0.98–1.06)	31.5 °C (Q94)	1.00 (0.99–1.02)	1.03 (0.99–1.06)
Natural causes	28.2 °C (Q90)	1.00 (0.98–1.02)	**1.05 (1.00–1.09**)	30.5 °C (Q90)	0.99 (0.98–1.01)	**1.05 (1.01–1.09**)
65+						
Alzheimer and dementia	27.3 °C (Q84)	1.00 (0.96–1.04)	**1.24 (1.11–1.38**)	28.2 °C (Q77)	1.04 (0.99–1.10)	**1.31 (1.18–1.45**)
Cerebrovascular diseases	28.0 °C (Q89)	1.00 (0.97–1.03)	**1.09 (1.02–1.17**)	29.2 °C (Q84)	1.01 (0.98–1.04)	**1.10 (1.03–1.17**)
Chronic lower respiratory diseases	-	1.01 (0.96–1.07)	1.04 (0.93–1.18)	-	1.00 (0.95–1.05)	1.04 (0.93–1.15)
Diabetes	28.2 °C (Q90)	1.00 (0.97–1.04)	**1.10 (1.01–1.21**)	29.5 °C (Q85)	1.02 (0.98–1.06)	**1.12 (1.03–1.21**)
Heart failure	-	0.99 (0.93–1.05)	0.98 (0.85–1.13)	-	1.00 (0.95–1.06)	1.02 (0.90–1.16)
Hypertensive diseases	27.3 °C (Q84)	1.03 (0.99–1.08)	**1.15 (1.05–1.26**)	27.0 °C (Q68)	**1.06 (1.02–1.10**)	**1.18 (1.08–1.28**)
Influenza and pneumonia	24.4 °C (Q54)	**1.04 (1.01–1.07**)	**1.08 (1.01–1.15**)	27.0 °C (Q68)	**1.04 (1.01–1.07**)	**1.11 (1.05–1.18**)
Ischemic heart disease	28.7 °C (Q93)	0.99 (0.96–1.01)	1.03 (0.97–1.10)	30.7 °C (Q92)	0.99 (0.97–1.02)	1.06 (1.00–1.12)
Malignant neoplasms	-	1.01 (0.99–1.03)	1.02 (0.97–1.07)	-	1.01 (0.99–1.03)	1.00 (0.96–1.04)
Other disease of the circulatory system	28.5 °C (Q92)	0.99 (0.96–1.02)	1.06 (0.98–1.14)	30.2 °C (Q89)	1.02 (0.99–1.06)	**1.08 (1.01–1.16**)
Other diseases of the respiratory system	27.5 °C (Q85)	1.01 (0.96–1.06)	**1.18 (1.04–1.33**)	30.0 °C (Q88)	0.99 (0.95–1.05)	**1.14 (1.02–1.27**)
Sepsis	29.2 °C (Q95)	1.02 (0.98–1.07)	1.05 (0.95–1.17)	-	1.04 (1.00–1.08)	1.06 (0.97–1.17)
Undetermined deaths	26.3 °C (Q74)	**1.06 (1.01–1.11**)	**1.37 (1.22–1.52**)	27.5 °C (Q72)	**1.08 (1.03–1.13**)	**1.42 (1.29–1.57**)
Urinary tract infections	26.1 °C (Q73)	**1.05 (1.01–1.09**)	**1.27 (1.15–1.41**)	27.2 °C (Q70)	**1.07 (1.04–1.11**)	**1.33 (1.22–1.44**)
Selected causes	25.9 °C (Q71)	**1.02 (1.01–1.03**)	**1.10 (1.07–1.13**)	27.0 °C (Q68)	**1.03 (1.02–1.04**)	**1.11 (1.08–1.14**)
Natural causes	26.1 °C (Q73)	**1.02 (1.01–1.04**)	**1.12 (1.09–1.16**)	27.5 °C (Q72)	**1.03 (1.01–1.04**)	**1.15 (1.11–1.18**)

Bold values indicate associations with 95% confidence intervals excluding the null value (1).

**Figure 2.**
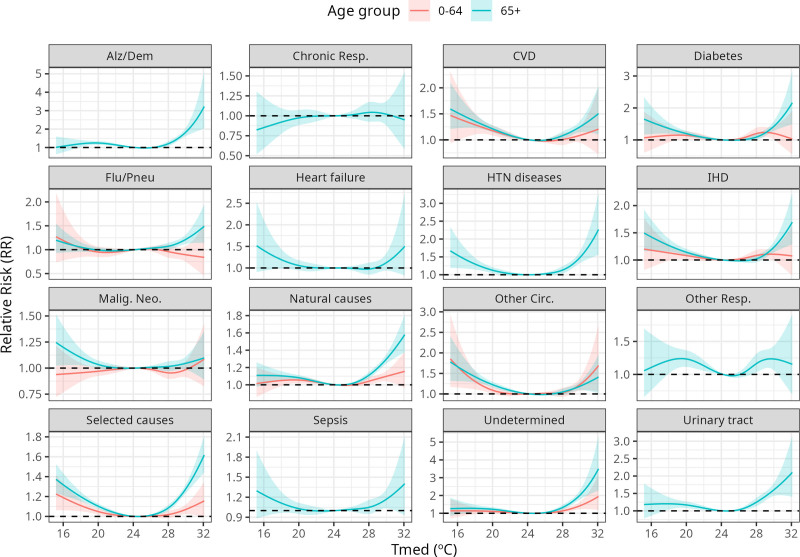
Average temperature (T_med_) effect on mortality (relative risk [RR]), all lags, by death cause and age group.

Figure 2 shows the exposure-response curves for T_med_, for all lags. Only results for T_med_ were shown because it showed lower AIC values than HI_med_ for most causes (see Model results section), but similar visualizations for HI_med_ can be seen in Supplementary Figure 8; https://links.lww.com/EE/A429. As expected, curves for the 65+ age group reach higher RR values than the younger one. For the latter group, significant and sustained increases in RR for heat are seen mostly for the aggregate causes (selected and natural), other diseases of the circulatory system, and undetermined causes. Among the elderly, standout groups (those that reach higher significant RR’s for heat) are Alzheimer’s and dementia, undetermined deaths (RR > 3 reached), hypertensive diseases, diabetes, and urinary tract infection (RR > 2 reached).

### Risk across lag dimension

**Figure 3.**
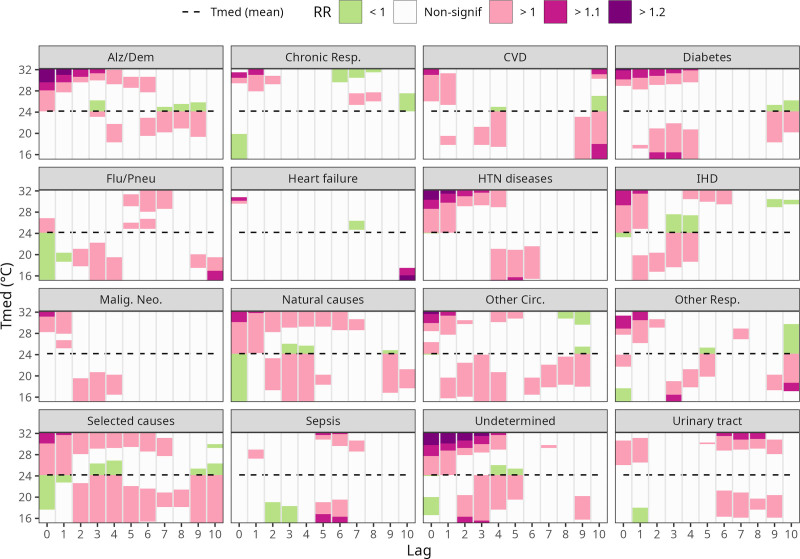
Average temperature (T_med_) effect on mortality (relative risk [RR]), by lag and death cause, for the 65+ age group.

In Figure 3, the same results for the T_med_ DLNM model are shown across the exposure and lag scales, by cause of death. Only results for the elderly (65+) are shown since they reached statistical significance for more causes, but Supplementary Figure S10; https://links.lww.com/EE/A429, shows the same results for the younger group. For the majority of causes, the standard pattern of a higher RR in earlier lags (0–2) and a subsequent decrease is seen. That is true for Alzheimer’s/dementia, diabetes, hypertensive diseases, IHD, and for natural, selected, and undetermined causes. Different patterns can be named: for influenza/pneumonia and sepsis, a significant RR for heat is seen in lags 5–7; and for urinary tract infections, higher RR for heat concentrates among lags 6–9.

### Hours of heat exposure

**Figure 4.**
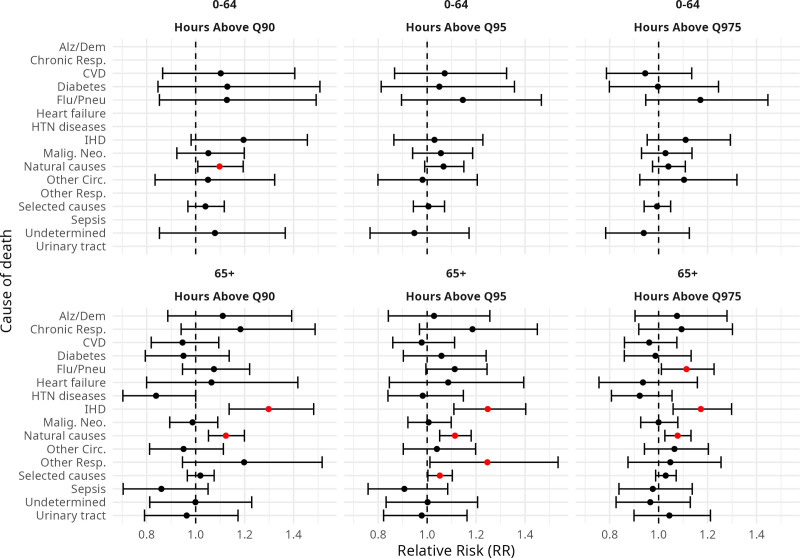
Additional effect of having a high amount of hours of heat index (HI) greater than its 90th quantile (Q90), 95th quantile (Q95) and 97.5th quantile (Q975) in the models already including average temperature (T_med_), by cause of death and age group.

The inclusion of the hours of exposure to high HI (Q90, Q95, and Q975) resulted in significant effects among few of the causes (Fig. 4). Among the 0–64 age group, hours above Q90 showed a significant RR estimate, but only for natural causes. For the elderly, significant estimates were reached for IHD (Q90, Q95, and Q975), other diseases of the respiratory system (Q95), influenza and pneumonia (Q975), natural (Q90, Q95, Q975), and selected causes (Q95). The highest RR was reached for IHD, for which an exposure to a high number of hours with HI above the Q90 percentile resulted in an increase of 30% in the mortality risk among the elderly, even after controlling for T_med_. For natural causes, a high number of hours above Q90 resulted in a 12% increase, and similar values of RR were obtained for the Q95 and Q975 variables (RR = 1.11 and RR = 1.10, respectively). Supplementary Figure S11; https://links.lww.com/EE/A429, shows similar results for models with the amount of hours + HI_med_.

### Model comparison

**Figure 5.**
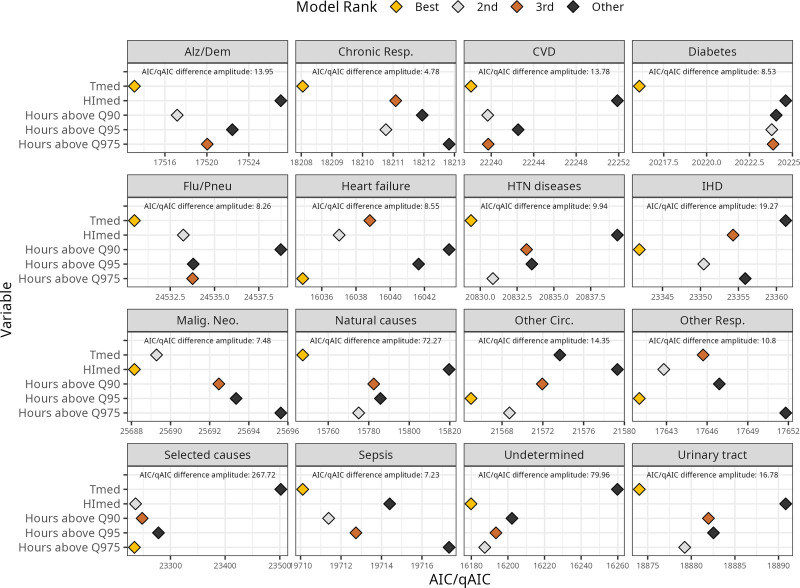
AIC (when Poisson) and qAIC (when quasipoisson) values for models for the elderly (65+) using different exposure variables: Daily average temperature (T_med_), daily average heat index (HI_med_), hours above HI 90th quantile (Hours above Q90), hours above HI 95th quantile (Hours above Q95), hours above HI 97.5th quantile (Hours above Q975).

Finally, Figure 5 exhibits AIC (when Poisson) or qAIC (when quasipoisson) obtained for models with T_med_ only, HI_med_ only, and T_med_ or HI_med_ with the added effects of number of hours (Q90, Q95, and Q975). The exhibited results are for the 65+ age group, while results for the younger group can be seen in Supplementary Figure S12; https://links.lww.com/EE/A429. Most causes had minimal difference in the AIC/qAIC when comparing best and worst performing models (<20). Highest differences occurred for selected causes (HI_med_: 23,236.15; T_med_: 23,501.80; difference of 256.65), undetermined causes (HI_med_: 16,179.84; T_med_: 16,259.80; difference of 79.96), and natural causes (T_med_: 15,747.40; HI_med_: 15,819.67; difference of 72.27). In general, T_med_-only models had lower AIC values 9 of the 16 modeled causes. HI_med_ performed best for malignant neoplasms and undetermined deaths; while models with added effects from hours of exposure had best results for 5 of the 16: heart failure, IHD, other diseases of the circulatory system, other diseases of the respiratory system, and selected causes.

## Discussion

This study evaluated the effect of heat exposure in mortality due to 17 causes and among two age groups, in RJC from January 2012 to June 2024. The studies with similar purposes found did not consider this amount of groups, nor covered the most recent period of important heat wave episodes in Brazil (November 2023 and January 2024). It also compares different metrics of heat exposure and the amount of exposure time simultaneously, by including the amount of hours above historically high HI thresholds.

### Death causes and age groups

As expected, fewer causes of death (six causes) showed significant heat-mortality associations for the younger group, when compared to the elderly (13 causes). Apart from the aggregate causes (selected and natural), only other circulatory diseases, diabetes, IHD, and unspecified causes presented a significant increase in RR for mortality in the young as T_med_ got higher (Fig. 2). Even though relevant RR marks were reached (1.21 for diabetes, 1.15 for undetermined deaths), CIs for the estimates tended to get wider in the extremes of the distributions. These findings are consistent with similar studies that show age as a considerable determinant in the heat-mortality relationship.^16,34,48^

Chronic lower respiratory diseases showed no association among the elderly, and other respiratory diseases also showed wide CIs. In addition to the low number of daily deaths observed for both causes (median of 3 for both), respiratory morbidity and mortality can be more pronounced in dry-hot heat conditions,^57^ which is less frequent in the region of the study.^58^ Moreover, the heat-mortality association for these causes is also known for interacting with air pollution^47,48^–which could not be considered in this study. Influenza and pneumonia, on the other hand, did present a significant association among the elderly–reaching higher RR values in lags 5–7 (Fig. 3).

The absence of association found for heart failure and sepsis can be justified by similar reasons. Both causes also have low death counts (≤4), and are also unspecific and usually not the underlying cause of death reported (variable upon which the study is based on). The ongoing recommendation by the Brazilian Ministry of Health is not to use nonspecific ICDs such as heart and respiratory failure or sepsis–so called “garbage codes”–as the underlying cause of death.^59^ Finally, even though higher mortality due to neoplasms during heat waves has been reported in the literature^60–62^ and majorly attributed to harvesting effect,^61^ we have not seen a significant effect in this study.

The heat-mortality relationship observed for IHD, cerebrovascular diseases (CVD), and other circulatory diseases in the elderly are consistent with the literature. The pathophysiological mechanisms in this relationship have been extensively described^12,23,63,64^ and involve a diminished capacity of heat stress responses, leading to higher cardiac demand and strain on the cardiovascular system. A recent meta-analysis including studies from more than 20 countries showed a 2.1% increase in cardiovascular mortality for every 1 °C increase in temperature, with cause-specific analyses showing higher risks for stroke, coronary heart disease, and heart failure.^63^ A cardiovascular effect was also seen in a study involving 326 Latin American cities^16^; and in studies that took place in Brazilian cities.^5,24,31,65^

Deaths due to diabetes also showed relevant associations with heat exposure. Among the elderly, a 10% increase in mortality was estimated for the 90th quantile of T_med_, and the RR even reaches values higher than 2 (double of the mortality baseline) for more extreme values of temperature (Fig. 2). Even though the relation of heat exposure with diabetes is still not totally clear, it is argued that diabetic patients have lower skin blood flow and thermoregulation mechanisms, which affect blood sugar control and cardiovascular regulation.^66^ Song et al,^66^ recent review of 18 studies, show a significant heat-mortality relation for diabetes, with a higher impact on mortality than morbidity. The strong association found in this study is supported by previous studies in Brazil: In a 2020 study in Rio de Janeiro Metropolitan Region, Geirinhas et al^33^ found that the highest excess mortality identified was due to diabetes, and particularly in women and the elderly. In addition to it, Zhao found a high association between heat wave episodes and hospitalization due to endocrine, nutritional, and metabolic diseases in Brazil during 2000–2015.^18^

One specific cause group that draws attention due to the high values of RR reached is mortality by Alzheimer’s and dementia. Even though daily counts for deaths due to this cause are low (median = 3), the estimated RR reached values higher than 3 in the extremes of the T_med_ distribution (Fig. 3), meaning a 200% increase is expected in that scenario. A higher number of deaths is expected shortly after the heat exposure, and up until 3 days later (Fig. 3). Alzheimer’s disease admissions had already been shown to be related to heatwave days.^67^ Xu et al,^68^ in a retrospective cohort study in Australia, reported an increased risk of hospitalizations due to Alzheimer’s disease during heatwaves, and reviewed plausible mechanisms, which include a change in thermoregulation capacity due to the use of neuroleptics; a dopamine deficit during heatwave days; and a mixed capacity of recognizing hostile environments on extreme temperatures.

Deaths due to urinary tract infections also showed significant associations for the elderly (RR = 1.37 for T_med_ Q90, RR > 2 in T_med_ extremes). Heat effects on renal and urological outcomes–which include kidney disease, renal failure, urolithiasis and urinary tract infections–have been documented in studies occurring in Australia^19,69^ and in American cities.^70,71^ In Brazil, Zhao et al^18^ also reported strong associations between severe heatwaves and admissions by genito-urinary causes. Heat exposure raises the potential of dehydration, which is already critical in the elderly.^72^ This reduces blood volume and urine output, which can impair renal hypoperfusion and lead to kidney injury, or increase the risk of urinary tract infections. One specificity to this cause group is that it was the one that reached high values of RR for longer lags–a higher RR estimate was observed roughly one week after the exposure (lags 6–9), which can be expected, but differs from another study in Brazil.^53,73^

Finally, unspecified, selected, and natural causes showed significant associations among both age groups. Highest RR estimates were obtained for unspecified (RR = 1.37 and RR = 1.15 for the 65+ and 0–64 age groups, respectively), followed by natural and selected causes. This indicates an existing burden of heat-related excess mortality, irrespective of age, when considered collectively. The fact that the highest excess occurs among the unspecified causes suggests a potential excess use of garbage codes, particularly in periods when there is a mortality excess during heat exposure days, which needs a deeper look.

### T_med_, HI_med_, and exposure period to heat as a metric for explaining heat-related mortality

Similarly to what has been observed in existing literature,^74–76^ the use of a composite index of temperature and humidity–such as the HI–showed no relevant benefit on the explanation of heat-related mortality, based on the AIC/qAIC. For most causes, little difference in such metric was observed among the models (Fig. 5 and Supplementary Figure S12; https://links.lww.com/EE/A429). The exceptions occurred among natural causes–for which T_med_ performed better–and selected and undetermined causes–for which HI_med_ showed better results. Therefore, no robust evidence favors one metric or another. As seen in exploratory visualizations, the HI captures more extreme days, which can aid the detection of abnormal heat exposure, but also brings more uncertainty in the estimation of the response curves.

Another aspect this study attempted to investigate is whether there is any additional risk depending on the amount of time exposed to high HIs in a given day. Even though daily summary metrics like T_med_, T_min_, or T_max_ have been consistently used in heat-mortality studies, they may not represent the cumulative exposure time within a day. Given that days with different exposure times to heat can have similar T_med_ values (Supplementary Figure S4; https://links.lww.com/EE/A429), the addition of an indicator variable showing whether a day had longer periods of exposure to heat was tested. Even though these indicator variables showed no additional contribution to explaining mortality across most causes, a relevant additional effect was seen for the more numerous groups: selected and natural causes. This implies that there is a difference in risk between days with similar daily averages but different exposure periods to extreme heat. These findings lend support to the concept that heat protocols, such as Rio’s employ the exposure periods to define its triggers. However, further research is needed to understand how to exactly quantify and incorporate this dimension.

### Limitations and future work

This study prioritized studying the association of heat exposure with specific mortality causes in the most recent years in Rio de Janeiro. Disaggregating by cause groups led to low death counts, which made difficult the consideration of broader age groups, sex, race, or socio/spatial differences in the analyses, even though evidence shows they play a significant role in the heat-related mortality.^5,25,26,77^ Pregnancy and maternal outcomes are also stated as related to heat exposure in the literature,^21,22^ and must be investigated in further studies for the RJC population.

Spatial differences in heat exposure and its related outcomes were overlooked. RJC is a large, urban city, prone to the urban heat island effect,^78^ where densely populated and urban areas, as well as those with lower vegetation cover, tend to accumulate more heat.^79^ These areas are also highly correlated with unfavorable socioeconomic conditions.^27,80^ Moreover, gaps in weather stations’ data also impose a challenge: many densely populated areas of the city lack station coverage (Supplementary Figure S1; https://links.lww.com/EE/A429), while part of the stations are located in airports or coastal locations, which do not capture intraurban heat heterogeneities.^75^

Final considerations on study limitations regard the complex role humidity plays on heat-related mortality. Even though it is clear that humidity strongly affects heat-related outcomes in human physiological studies,^11,81^ this effect is not clear in population studies.^75^ Moist heat affects individuals’ capacity to dissipate heat and therefore to cool themselves. This effect, when present for a large exposure time, can lead to heat exhaustion or heat stroke, particularly among the most vulnerable.^26,82^ However, epidemiological studies conducted considering humidity along with temperature^74^ or through a composite index,^83–86^ have shown little or no impact on explaining mortality. Our findings contribute to this trend, since models for HI_med_ had similar AIC/qAIC than T_med_ for most causes (Fig. 5). Baldwin et al^75^ contribute by pointing out the fragility of using such composite indices, since increases can be observed due to elevations in temperature, in humidity, or in both. Including both temperature and humidity (preferably, not relative humidity) in the mortality models–possibly, with an interaction or effect modification term–is encouraged and could help better understand how T and H relate to each of the mortality causes.

### Final considerations

Exposure to high heat levels for prolonged periods leads to an increase in mortality in Rio de Janeiro, primarily affecting the elderly population. Younger age groups are affected in smaller proportion and in a fewer number of causes, while diabetes, Alzheimer’s and dementia, urinary tract disorders, and hypertensive diseases stood as particularly affected causes among the elderly. The inclusion of indicator variables for days with a prolonged number of hours of extreme heat provided novel evidence of an additional effect related to the duration of the exposure. This approach innovatively highlights the importance of accounting for the exposure period in heat-health studies, and may aid the development of more specific thresholds for HWSs.

## Conflicts of interest statement

The authors declare that they have no conflicts of interest with regard to the content of this report.

## ACKNOWLEDGMENTS

*We would like to thank Rio’s Municipal Health Secretariat (SMS), Operations and Resilience Center (COR), Alerta Rio, and Municipal Secretariat for Public Services and the Environment (SMAC) for collaborative work.*

## Supplementary Material
