# Supplementary material for: Cause-specific heat-related mortality in Rio de Janeiro city: Comparing exposure metrics and the role of exposure duration
Source: Environ Epidemiol. 2026 May 28;10(3):e473. doi: 10.1097/EE9.0000000000000473 (PMC13221122; doi:10.1097/EE9.0000000000000473)
Supplement: Supplementary file 1 [file ee9-10-e473-s001.pdf]

<u>**Supplementary Material**</u>

*Cause-specific heat-related mortality in Rio de Janeiro city: comparing exposure metrics and the role of exposure duration*

**Figure S1.** Meteorological stations locations, for each of the three data sources included in the study.

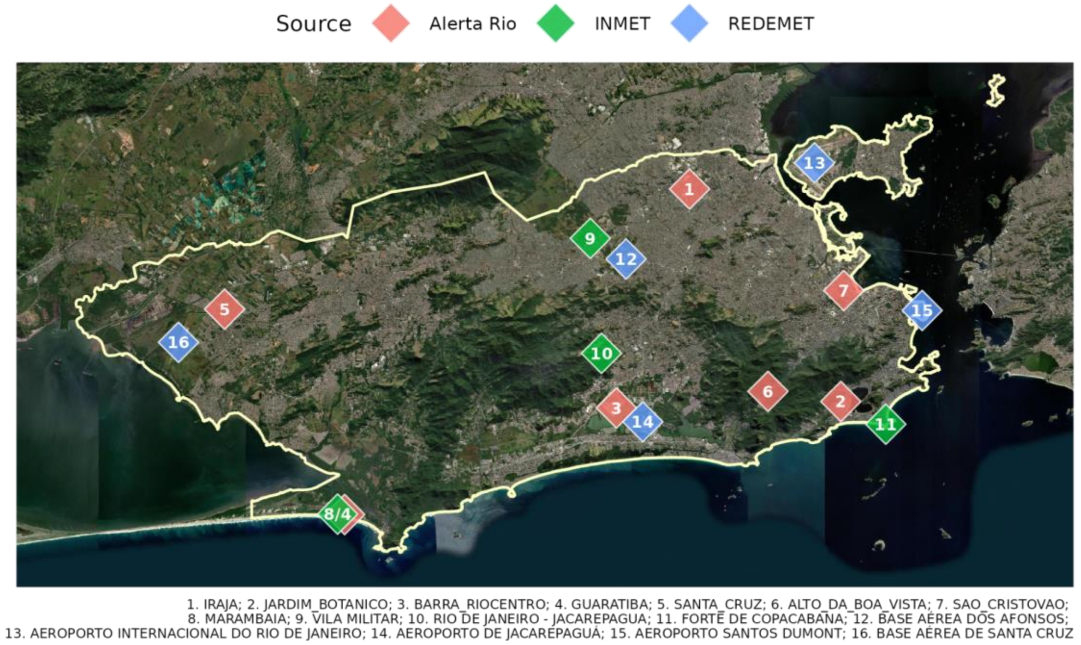


**Figure S2.** Distribution of daily death counts, by cause of death and age group.

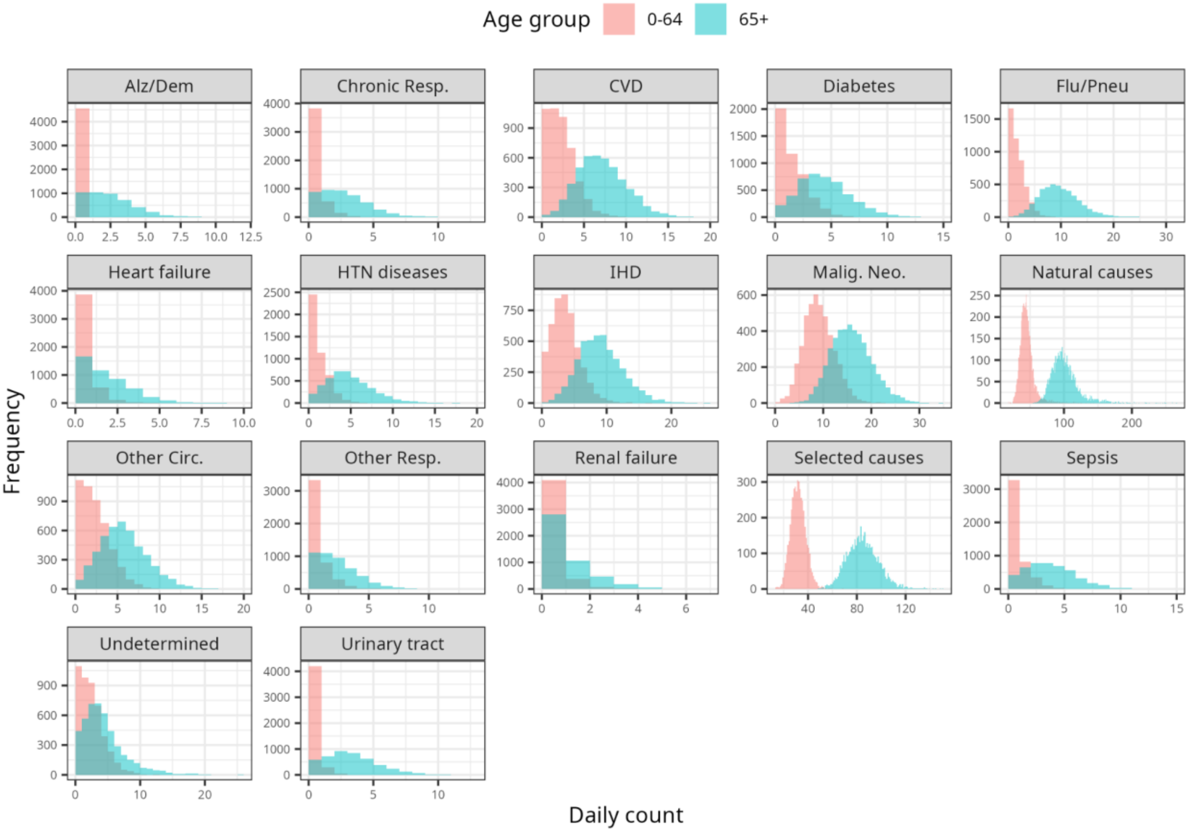

**Figure S3.** Distribution of $T_{med}$ and $HI_{med}$.

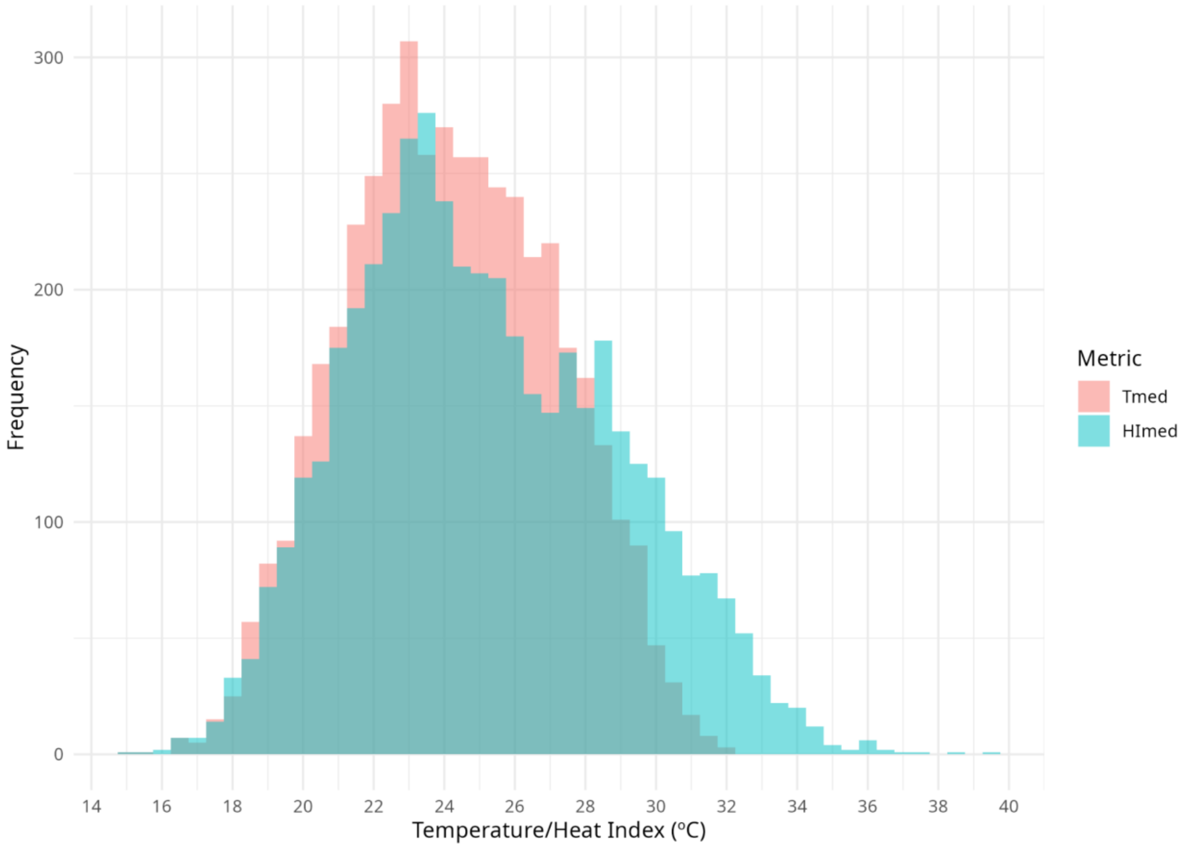


**Figure S4.** Comparison between $T_{med}$ values and amount of hours with heat index (HI) values above its 90th quantile within a day

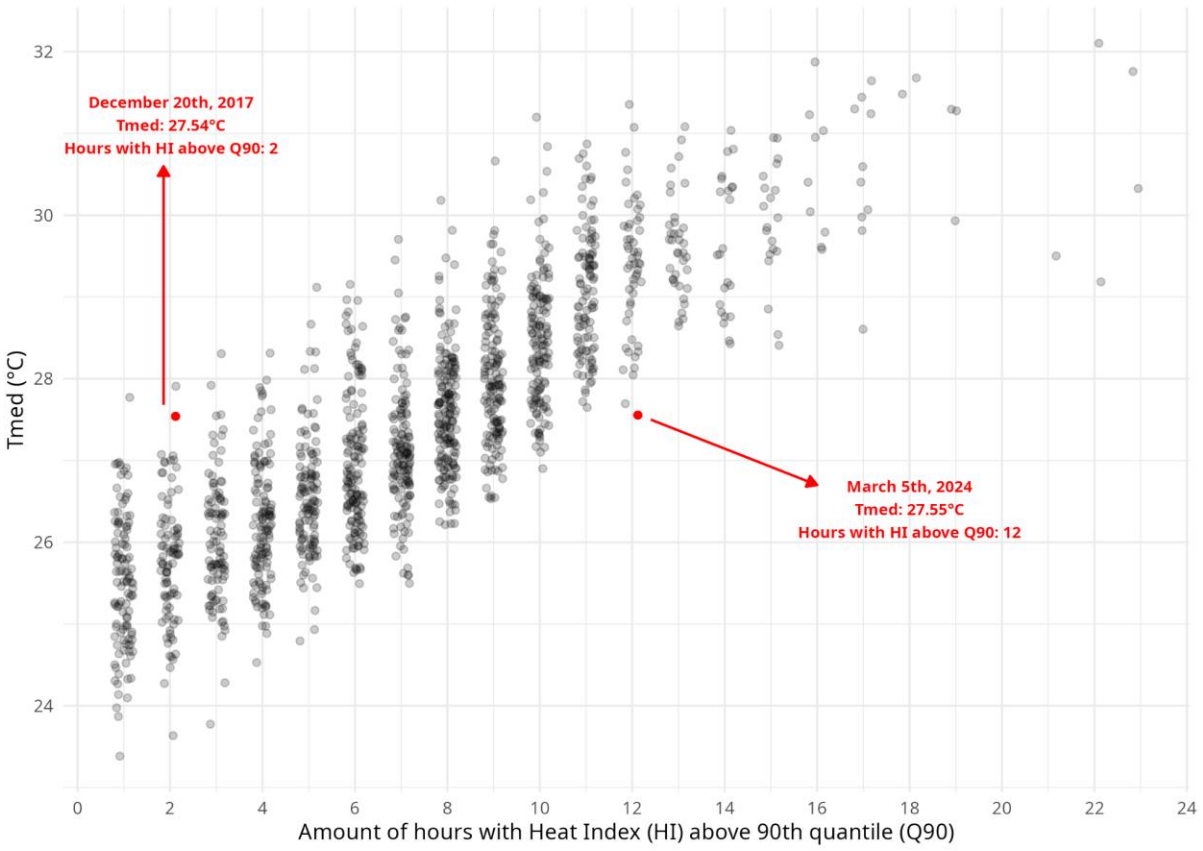


**Figure S5.** Estimated trend coefficient, by cause of death and age group.

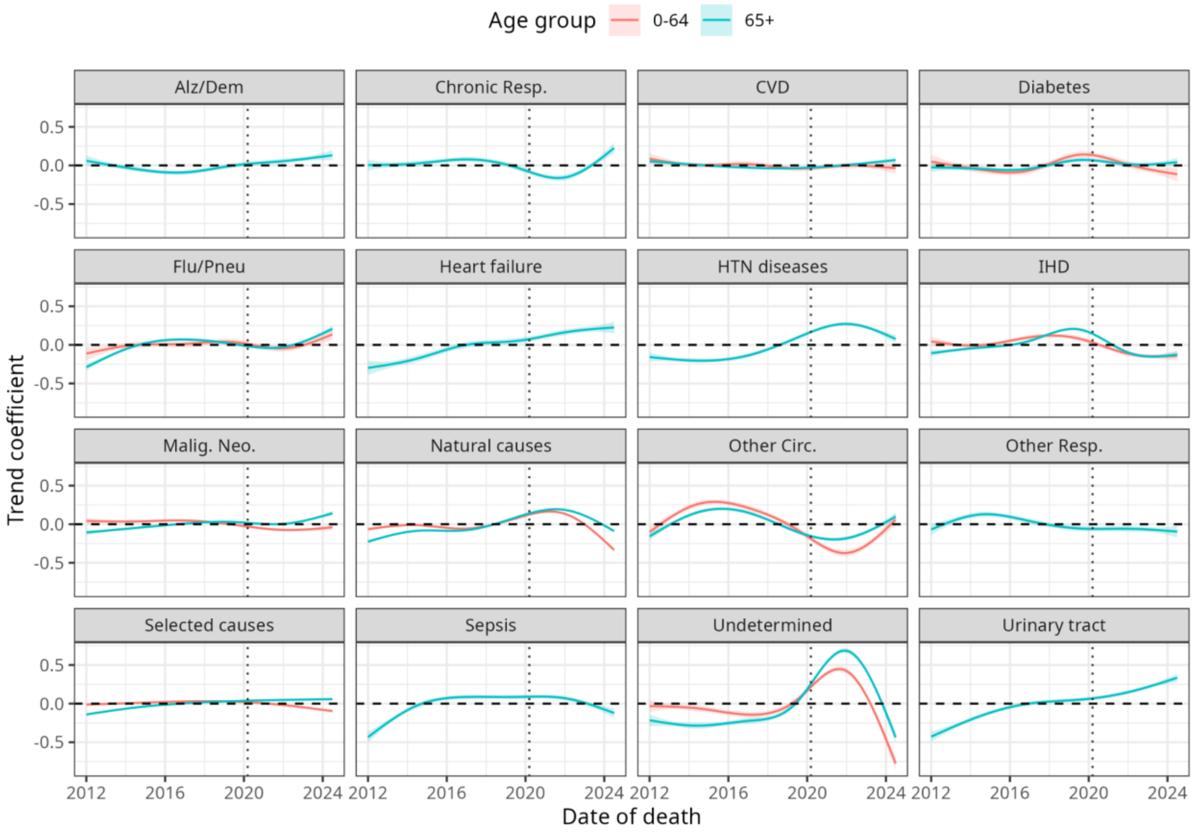

**Figure S6.** Estimated seasonality coefficient, by cause of death and age group.

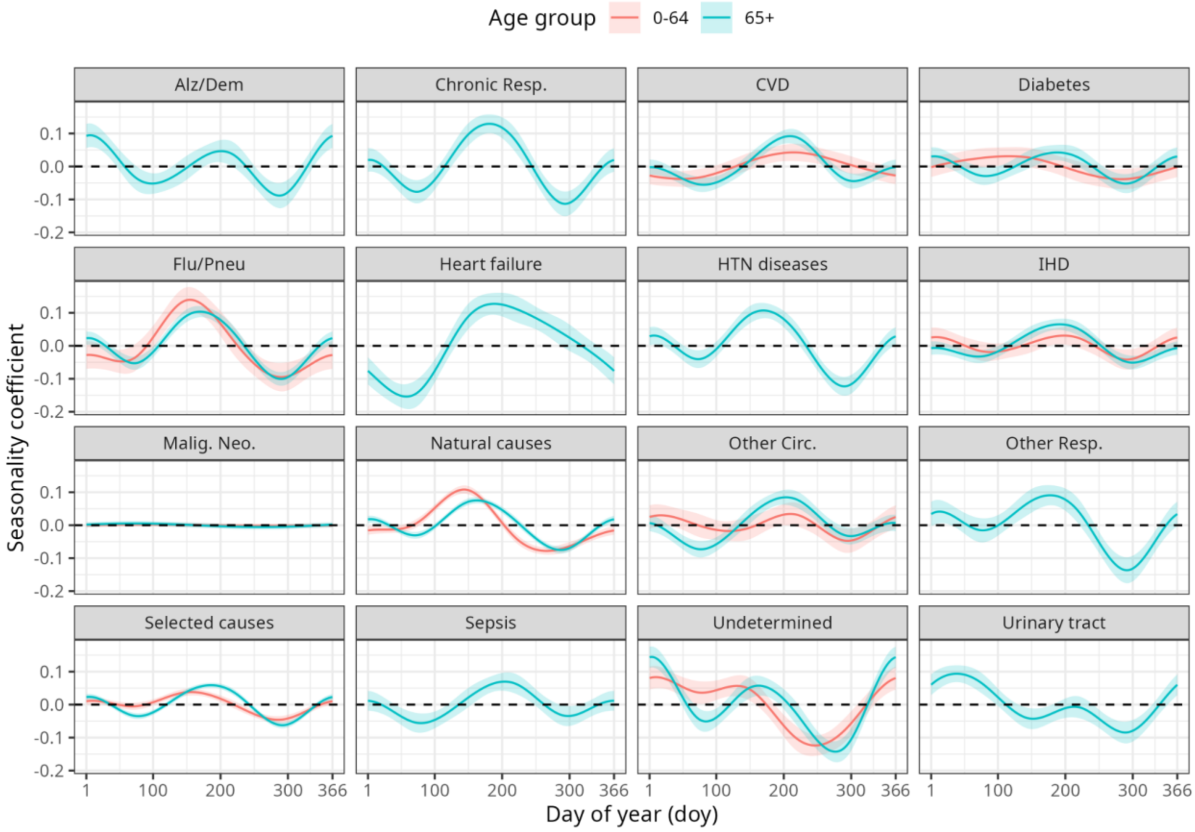


**Figure S7.** Estimated day of week (dow) coefficient, by cause of death and age group.

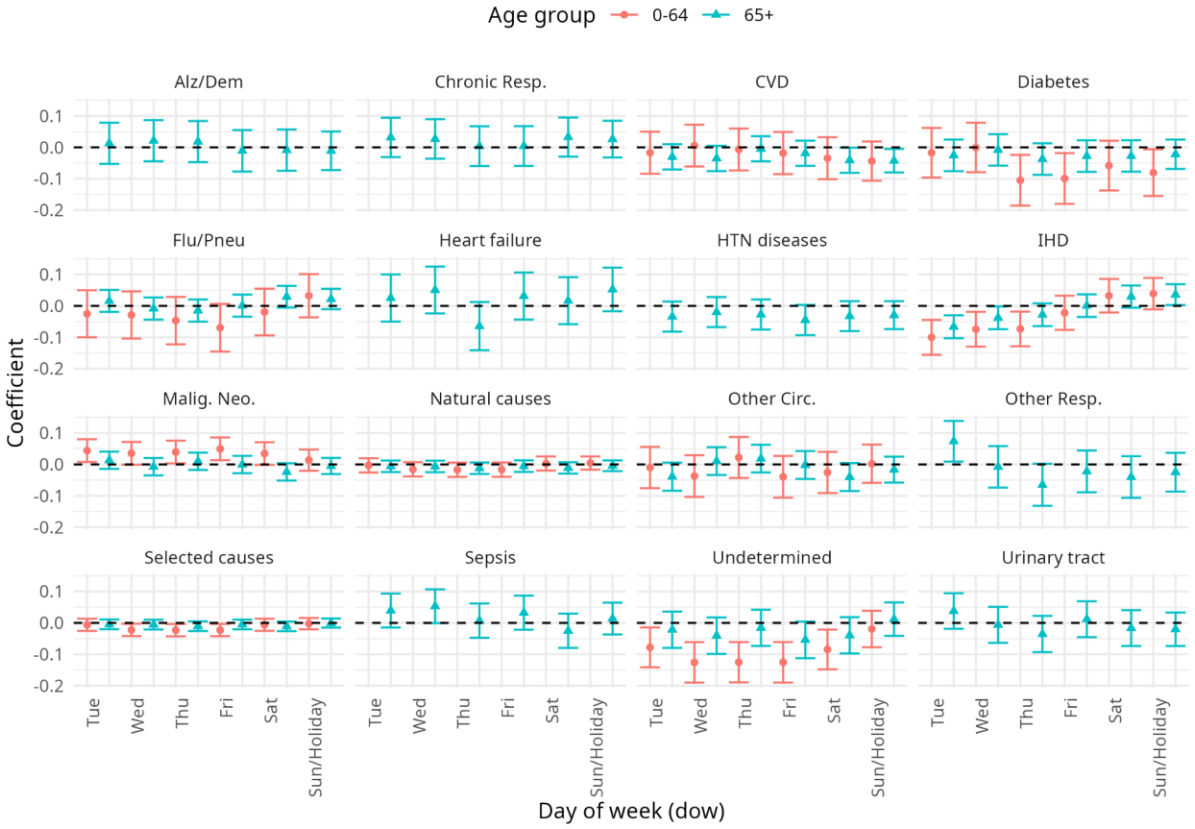

**Figure S8.** Average heat index (HImed) effect on mortality (Relative Risk - RR), all lags, by death cause and age group.

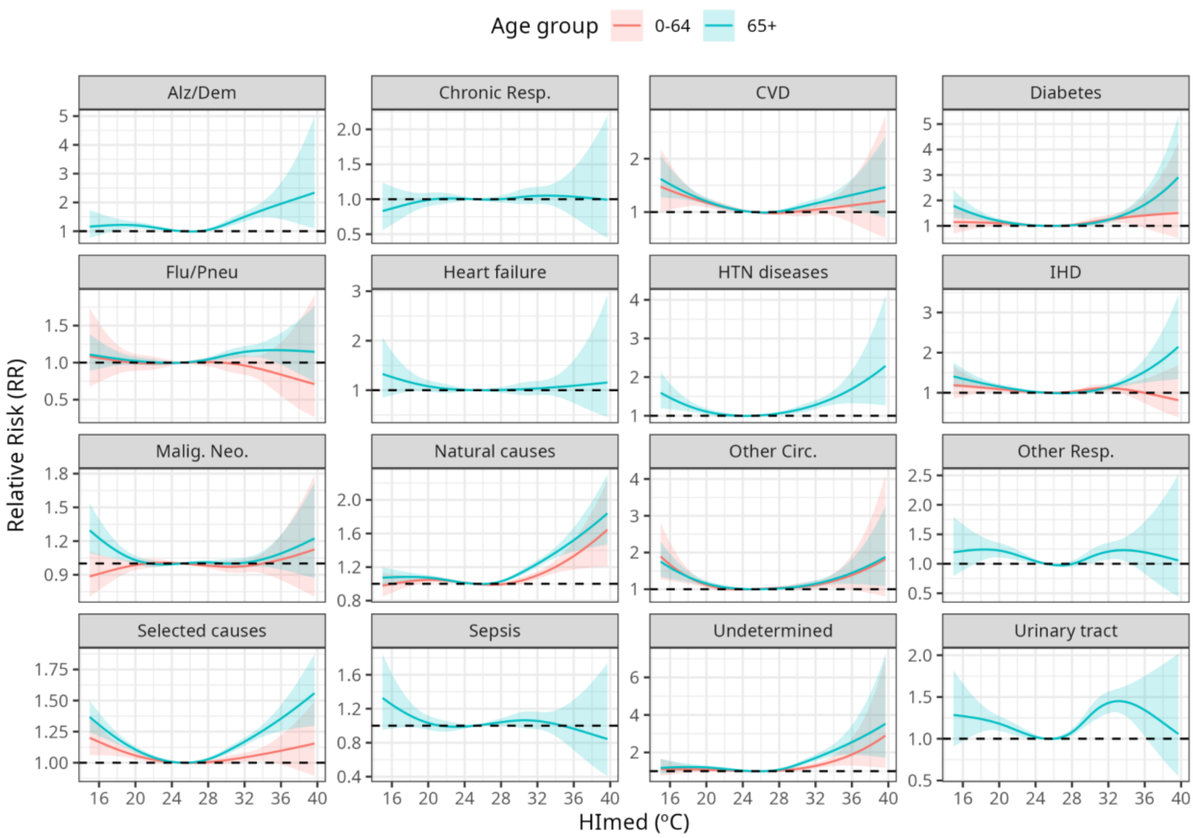


**Figure S9.** Estimated relative risks (RR's) for $T_{med}$ and $HI_{med}$, among the elderly (65+), by cause of death.

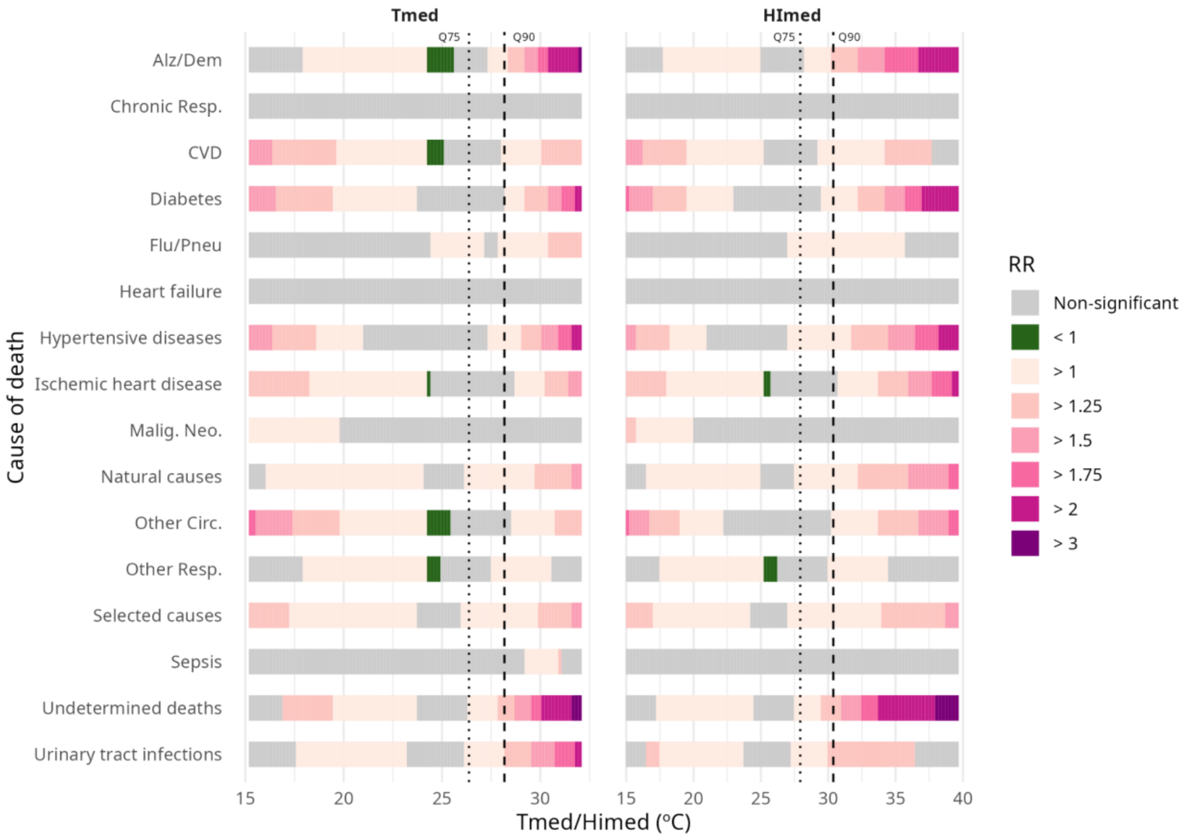


**Figure S10.** Average heat index (HImed) effect on mortality (Relative Risk - RR), by lag and death cause, for the 65+ age group.

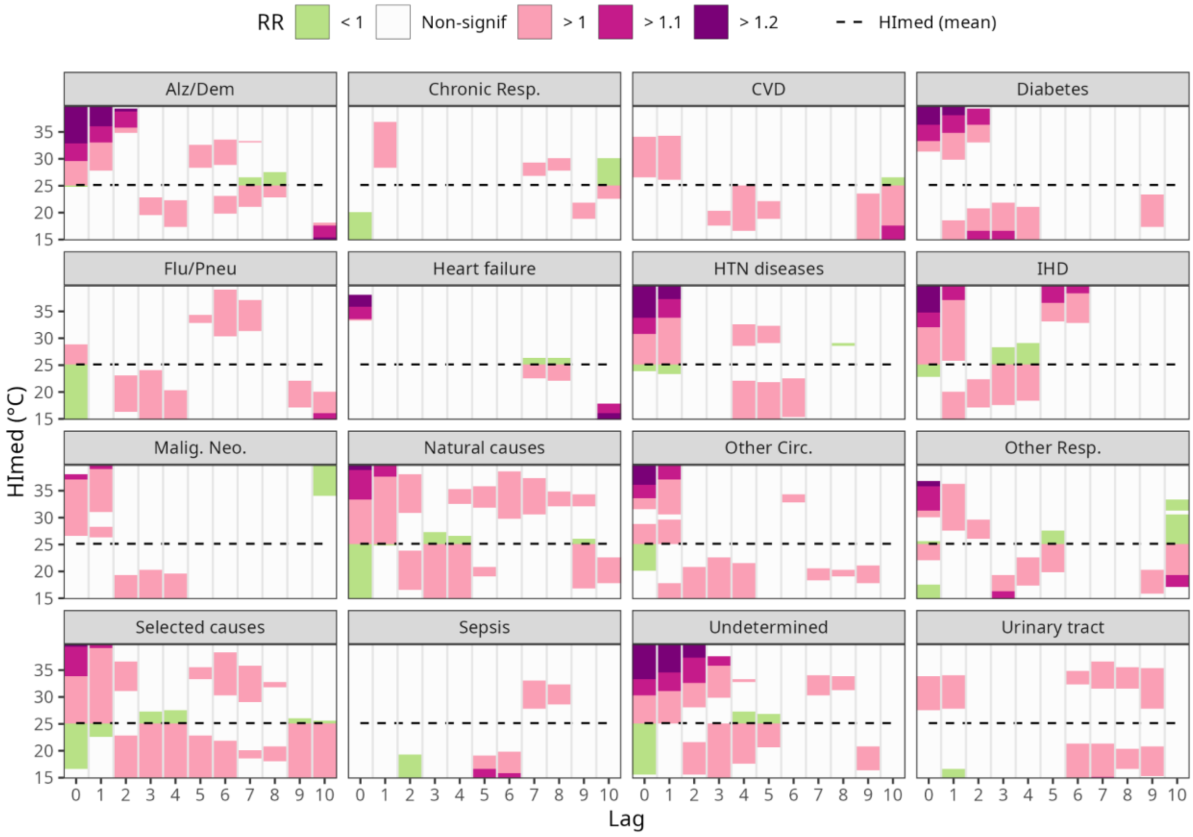

**Figure S11.** Additional effect of having a high amount of hours of heat index (HI) greater than its 90th quantile (Q90), 95th quantile (Q95) and 97.5th quantile (Q975) in the models already including average heat index (HImed), by cause of death and age group.

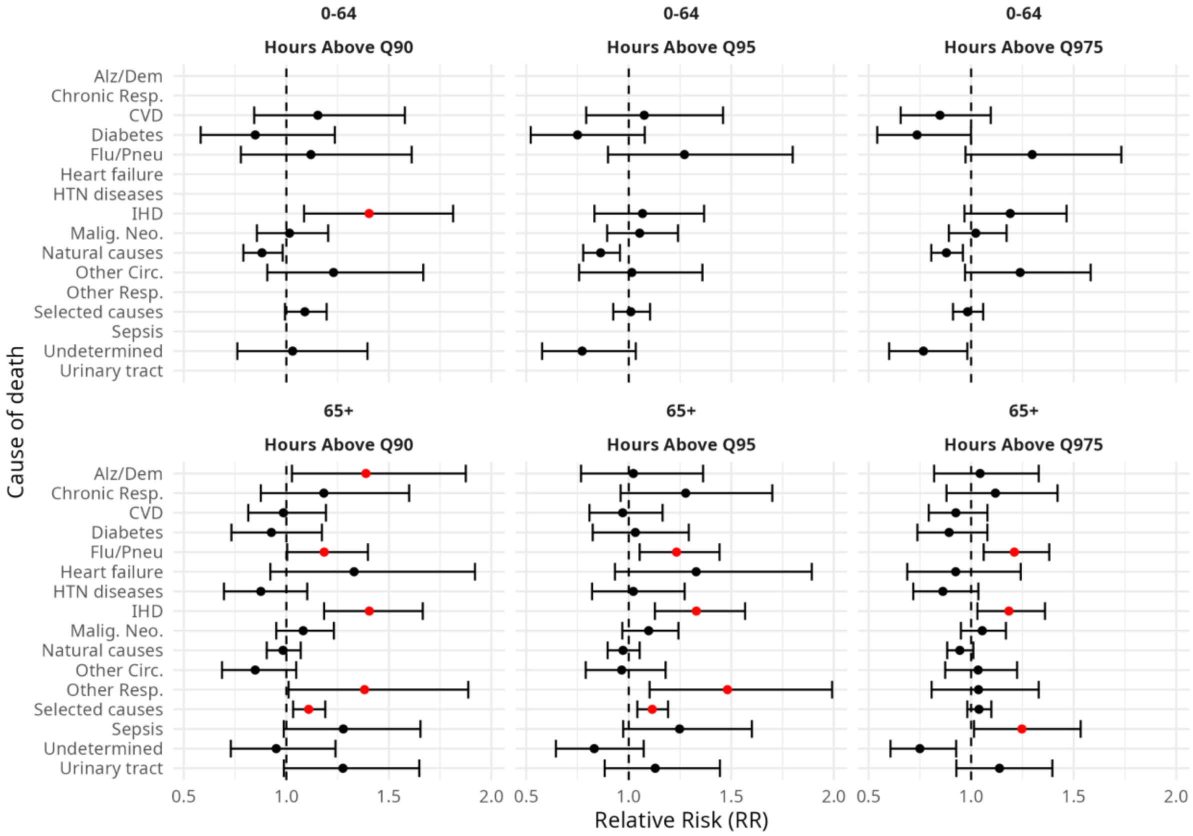


**Figure S12.** AIC (when Poisson) and qAIC (when quasipoisson) values for models for the 0-64 age group using different exposure variables: Daily average temperature (Tmed), daily average heat index (HImed), hours above HI 90th quantile (Hours above Q90), hours above HI 95th quantile (Hours above Q95), hours above HI 97.5th quantile (Hours above Q975).

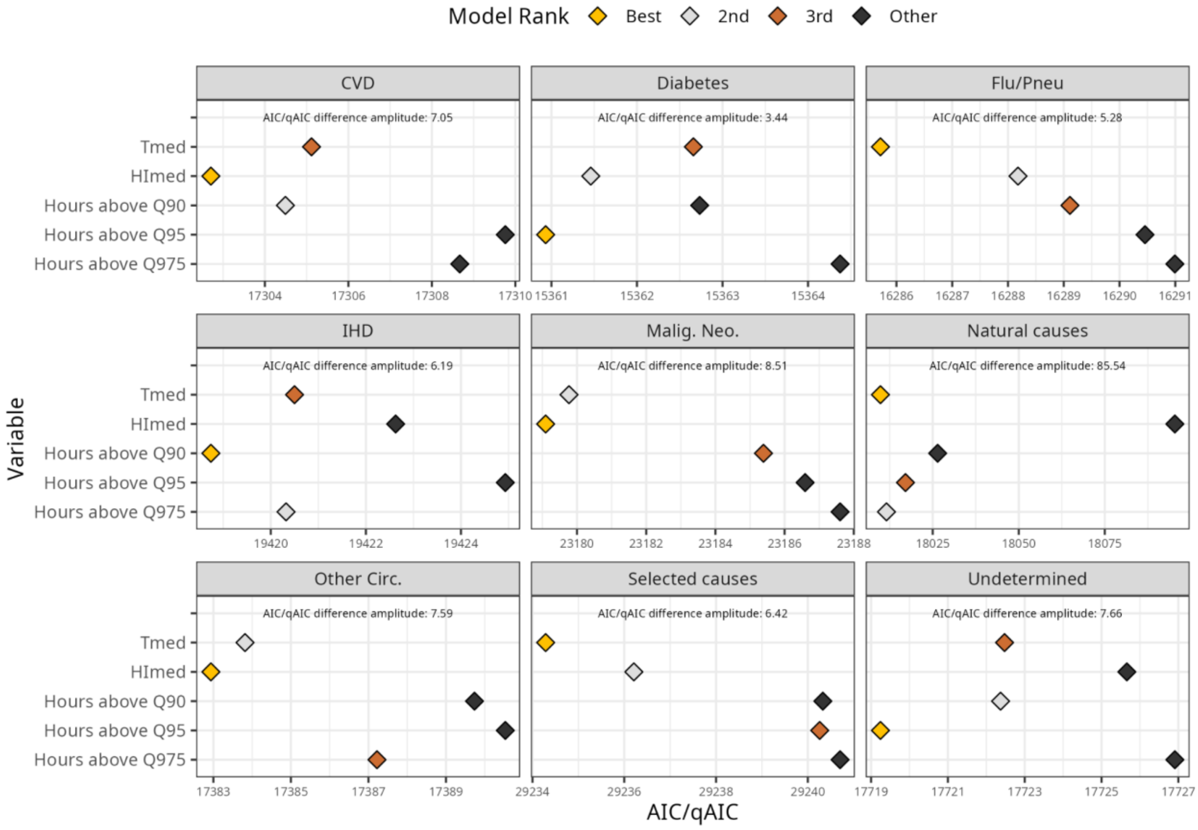

Model Rank
Best
2nd
3rd
Other
CVD
Diabetes
Flu/Pneu
IHD
Malig. Neo.
Natural causes
Other Circ.
Selected causes
Undetermined
AIC/qAIC difference amplitude: 7.05
AIC/qAIC difference amplitude: 3.44
AIC/qAIC difference amplitude: 5.28
AIC/qAIC difference amplitude: 6.19
AIC/qAIC difference amplitude: 8.51
AIC/qAIC difference amplitude: 85.54
AIC/qAIC difference amplitude: 7.59
AIC/qAIC difference amplitude: 6.42
AIC/qAIC difference amplitude: 7.66
Tmed
HImed
Hours above Q90
Hours above Q95
Hours above Q975
Variable
AIC/qAIC
